# The Antioxidant Potential of Black Tea Polyphenols in Heavy Metal Toxicity: An In Vitro Perspective

**DOI:** 10.3390/ijms26167926

**Published:** 2025-08-16

**Authors:** Ewa Wnuk

**Affiliations:** Department of Biomedicine and Environmental Research, Institute of Biological Sciences, Faculty of Medicine, The John Paul II Catholic University of Lublin, Konstantynow Ave. 1J, 20-708 Lublin, Poland; e.wnuk@kul.pl

**Keywords:** black tea, theaflavins, thearubigins, oxidative stress, heavy metals

## Abstract

Black tea is one of the most widely consumed beverages in the world, second only to water. Its properties are primarily due to the presence of polyphenols, which are associated with various effects, including antioxidant, anticancer, antiviral, and anti-inflammatory activities. These compounds include, among others, theaflavins (TFs) and thearubigins (TRs). The available literature provides numerous reports confirming the positive properties of these compounds. However, it is important to highlight their potential protective effects against oxidative stress induced by heavy metals. This is an important topic, given the ongoing exposure to such pollutants—resulting mainly from industrial development, among other factors. This manuscript provides a summary of the current knowledge on the properties of TFs and TRs and their effect against oxidative stress caused by heavy metals in in vitro studies. The limited amount of research in this area shows that this is still a poorly researched topic. Comparing the results obtained for epigallocatechin gallate (EGCG) from green tea, which has a similar structure to TFs and TRs suggests that they may exhibit similar or even enhanced antioxidant properties. Considering that black tea is consumed much more frequently than green tea worldwide, it is understandable that the antioxidant potential of these two compounds on cells needs to be investigated.

## 1. Introduction

Tea is the second most-consumed beverage in the world, after water; daily consumption is estimated at approximately 20 billion cups (1 cup = 200 mL) [1]. It is obtained from the tea plant *Camellia sinensis* (L.) Kuntze (Theaceae), which is primarily cultivated in Asia, with China and India being the most prominent producers. There are two main types of tea plant: *Camellia sinensis* var. *assamica* and *C. sisensis* var. *sinensis*. The former is characterised by big leaves and is used mainly in black tea production, whereas the latter, which has small leaves, is predominantly used in green tea production [2]. Bangladesh and India are considered the world’s largest producers of black tea, while Japan is the leading producer of green tea [3]. Taking global tea consumption into account, black tea is the most popular, accounting for approximately 78% of total tea consumption. Green tea ranks second, representing only 20%. The remaining 2% of worldwide consumption is attributed to oolong tea [2]. Several types of tea can be distinguished based on how they are produced. The most precise classification was proposed by Yi and colleagues [4], who identified as many as seven subtypes of tea: white, green, black, yellow, oolong, ripened Pu-erh, and aged Pu-erh. Green and white tea are non-fermented products in which the oxidation of compounds called catechins does not occur. As mentioned earlier, green tea is produced from young leaves and is therefore considered of high quality. Black tea is fully fermented, a process where the aerobic oxidation of tea polyphenols occurs [5,6].

Black tea’s fermentation stage distinguishes it from other teas in terms of taste, appearance, and chemical composition. Furthermore, factors such as the type and age of the leaves, as well as the climate and agricultural practices used, also influence the composition of tea. Due to the production process, the main differences between tea types are their catechin content and levels of oxidised polyphenols. Using green and black tea as examples, the amount of catechins in green tea is approximately three times higher than in black tea (30–35% and 3–10%, respectively), whereas the level of oxidised polyphenols in black tea is about four times higher than in green tea (23–25% and 6%, respectively) [7] (Table 1). In black tea, almost 75% of catechins are transformed through an aerobic oxidation process [1]. The most important result of this transformation is the formation of theaflavins (TFs) and thearubigins (TRs) [6].

Compared to green tea, whose health-promoting properties have been widely researched and described, the properties of black tea still require in-depth investigation. One of the main reasons for this is the difficulty in the detection of TFs and TRs in urine and blood. Nevertheless, some studies have reported increases in catechin levels following black tea ingestion [8]. The antioxidant properties of black tea have been the focus of several studies [9]. It is believed that TFs and TRs are responsible for the antioxidative potential of this beverage. TFs have also been reported to exhibit stronger antioxidative properties than glutathione, tocopherol or ascorbic acid [10]. Notably, black tea polyphenols are capable of forming complexes with iron or copper ions, which prevents the generation of free radicals and inhibits lipid peroxidation [11].

This review aims to present the two main groups of antioxidant compounds found in black tea—theaflavins (TFs) and thearubigins (TRs)—and to evaluate existing in vitro studies that investigate their effects on heavy metal toxicity.

### 1.1. Theaflavins

**Figure 1:**
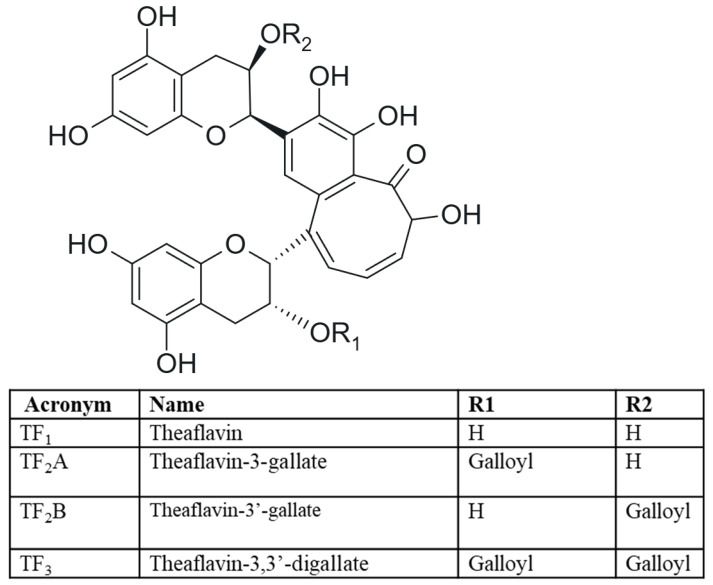
Structures of theaflavins.

Theaflavins (TFs) are among the major components of black tea, accounting for approximately 2–6% of its dry weight. These orange pigments are responsible for the tea’s bitter, strong flavour and dark colour [1]. Due to their biological and therapeutic properties, theaflavins are often referred to as “golden molecules” [12]. Theaflavins are formed through a process catalysed by polyphenol oxidase (PPO) and peroxidase (POD), involving the dimerization of epicatechins found in green tea: epicatechin (EC), epigallocatechin (EGC), epicatechin gallate (ECG) and epigallocatechin gallate (EGCG). In black tea, four main types of theaflavins can be identified: theaflavin (TF_1_ formed from EC and EGC), theaflavin-3-gallate (TF_2_A, ECG and EGC), theaflavin-3′-gallate (TF_2_B, EC and EGCG) and theaflavin-3,3′-gallate (TF_3_, ECG and EGCG) [2,13] (Figure 1).

Theaflavins are known for their numerous beneficial effects. One of the most frequently cited properties is their strong anti-oxidative activity. As oxidation products of polyphenols such as EGCG, which itself exhibits anti-oxidative activity, TFs also retain this function. Considering the individual types of TFs, their antioxidant activity increases in the following order: TF1 < EGCG < TF2A = TF2B < TF3 [12,14,15]. The properties are attributed to the specific structure of TFs. The benzotropolone skeleton, formed during co-oxidation and coupling of specific catechins, provides a structure that offers more sites for interaction with free radicals. Numerous studies have shown that having a greater number of OH groups in TFs, compared to other catechins, contributed to significantly higher antioxidant activity than that of EGCG [12,16,17]. The prevention of oxidative damage caused by lipid peroxidation is possible due to the ability of TFs to scavenge superoxide anions and chelate metal ions [18,19,20]. TFs have been shown to protect PC12 cells treated with H_2_O_2_ from oxidative stress, as evidenced by increased cell viability and downregulation of Bax/Bcl-2 and caspase-3 expression [21]. It has been shown that TFs also exhibit antimutagenic properties, can inhibit signal transduction during cell proliferation, prevent H_2_O_2_ and t-BuOOH (tert-butyl hydroperoxide)-induced cytotoxicity, and reduce oxidative stress and DNA damage [22].

The anti-carcinogenic properties of TFs have also been investigated. They significantly influence the proliferation, migration, and apoptosis of various cancer cells types [23]. Studies using mammary epithelial carcinoma, human breast cancer, and human prostate cells as models reported increased levels of Bax, caspase-3 and caspase-9, as well as decreased expression of Bcl-2–findings that suggest the induction of cell apoptosis [24,25]. The inhibition of MAPK and Akt survival pathways by theaflavins has also been observed [25,26]. Furthermore, it has been shown that TFs inhibit the growth of lung cancer lines H441, H661 and H1299 [27].

Theaflavins exhibit inhibitory activity against several viruses. They have been shown to inhibit the replication of influenza viruses [28] and coronaviruses [29], possess anti-rotavirus (RV) activity [30], inhibit the hepatitis C virus (HCV) by blocking viral DNA replication [31], and suppress HIV entry to cells [32].

The anti-inflammatory properties of TFs should also be included among their previously mentioned biological activities. Zu and colleagues [33] observed decreased IL-6 expression under the influence of TFs, which is particularly significant given the role of IL-6 in tissue injury and cell apoptosis. Moreover, Gosslau and colleagues reported the suppression of 12-O-tetradecanoylphorbol-13-acetate-induced COX-2 gene expression, as well as downregulation of NF-ĸB and TNF- α signalling factors [34].

Despite their numerous advantages, the ability to produce H_2_O_2_ and induce apoptosis has also been reported. Yang et al. (2000) observed earlier and more rapid cell death in 21BES cells (Ha-ras gene transformed human bronchial epithelial cell lines) compared to cells treated with 25 µM EGC or EGCG for 24 h [27].

### 1.2. Thearubigins

**Figure 2:**
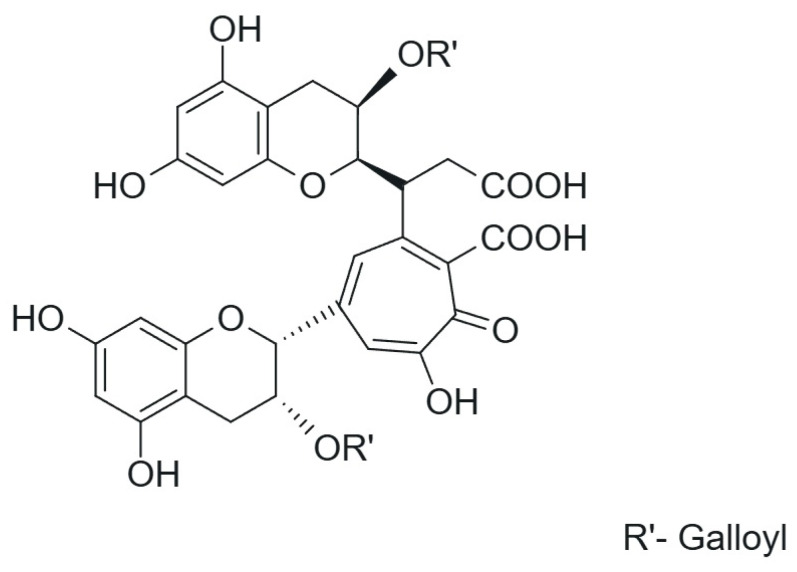
Proposed structure of thearubigins.

Unlike TFs, which have been well characterised and described over the years, the exact properties and characteristics of thearubigins (TRs) still require further analysis. A major challenge is that TRs are very difficult to isolate under laboratory conditions. It is known that TRs comprise a mixture of substances with molecular weights ranging from 1000 to 40,000 [35]. The TR content in black tea is higher than that of TF, at approximately 12–16%, making them the most abundant group of compounds in black tea [1,13]. They are characterised with a red-brown or dark brown colour and are believed to be responsible for the red-brown appearance of tea and its “heavy” taste [36]. TRs are formed during the PPO- and POD-catalysed oxidation of TFs and catechins [37]. It is hypothesised that they are heterogeneous flavan-3-ols and their gallate derivatives (Figure 2).

Concerning the properties of TRs, the available literature is quite scarce in terms of reports on their positive or negative effects. Similarly to TFs, TRs have demonstrated antioxidative properties. They exhibit inhibitory effects on lipid peroxidation, as shown by Yoshino et al. [10] in liver tissue suspensions. Furthermore, they protected HPF-1 cells from oxidative stress induced by H_2_O_2_ [15]. It has also been concluded that TRs can inhibit the formation of thiobarbituric acid reactive substances (TBARS) and Cu-induced lipid peroxidation of low- and high-density lipoproteins (LDL and HDL). The antioxidant activity of TRs is attributed to their ability to suppress the formation and accumulation of intracellular reactive oxygen species (ROS) and to inhibit peroxyl radicals by preventing membrane lipid oxidation [38].

In addition to their antioxidant properties, TRs—like TFs—are also characterised by anticancer activity. Imran et al. [39] observed reduced cell viability in colon cancer (HCT 116) and lung cancer (HT 560) cell lines following exposure to TRs. Moreover, flow cytometry revealed substantial cell cycle arrest at the G2/M phase. Notably, the effect on these cells was significantly stronger when TRs were combined with TFs. Similar conclusions were reached by Bhattacharya and colleagues in their studies. In their first study, it was concluded that TRs induce apoptosis of human malignant melanoma cells (A375) via the MAPK kinase pathway and the JNK–p38 cascade, triggered by intracellular oxidative stress [26]. In their second study, the anticancer activity of TRs was examined in human leukemic cells (U937). The results confirmed cell apoptosis under the influence of TRs, with no adverse effects on normal human cells (peripheral blood mononuclear cells) at doses below 100 µg/mL [40]. Halder et al. [41] also investigated the anticancer effects of TRs on A375 and A431 (human epidermoid carcinoma) cell lines. Their findings supported previous observations regarding apoptosis, cell cycle arrest, and the absence of toxicity on healthy cells. Furthermore, the analyses showed activation of caspase-9 and caspase-3, depolarisation of mitochondrial membrane potential, upregulation of p53 and p21, and stimulation of ROS generation following TRs administration.

Further confirmation of the antioxidant properties of TRs was provided by Wang and colleagues [42], who observed reduced levels of MDA, H_2_O_2_ and OH during neonatal acute lung injury (ALI). This study also demonstrated the anti-inflammatory properties of TRs, as a reduction in lung inflammation was observed, as a result of decreased cytokine levels in alveolar cavities.

### 1.3. Protective Effects of TFs and TRs

The protective efficacy of black tea polyphenols is heavily dependent on their bioavailability and metabolic conversion. TFs—including TF_1_, TF_2_A, TF_2_B, and T_3_—exhibit extremely low intestinal absorption, owing to instability of galloyl moieties and active efflux by P-glycoprotein (P-gp), multidrug resistance-associated proteins (MRPs), and breast cancer resistance protein (BCRP) transporters; these compounds are also metabolized into simpler phenolics such as gallic acid during trans-epithelial transport in Caco-2 cell models (study on mechanisms) [43].

Furthermore, human feeding trials and fecal fermentation studies have shown that colonic microbiota catabolize TFs into low-molecular-weight phenolic metabolites—phenyl-γ-valerolactones, hydroxyphenylpropionic acids, and gallic acid derivatives—that are detectable in urine and possess antioxidant and anti-inflammatory activity, suggesting that in vivo effects are mediated primarily by metabolites rather than parent TFs [44].

In contrast, TRs—as structurally complex compounds—are largely non-bioavailable intact and their physiological roles are inferred through microbial degradation pathways rather than direct absorption [45,46].

Consequently, the health-promoting actions attributed to TFs and TRs are largely dependent on their microbial biotransformation and resultant circulating metabolites, rather than native molecules.

## 2. The Effects of Heavy Metals on Oxidative Stress

Heavy metals (HMs) represent a serious threat to human health and life. Due to ongoing industrial development and other anthropogenic sources, humans are continuously exposed to these elements. Prolonged exposure to high concentrations of HMs can lead to various diseases, and in severe cases, even death. As a result, methods are constantly being sought to protect cells from the harmful effects of HMs or to reduce their toxicity. Cadmium (Cd), arsenic (As), lead (Pb) and mercury (Hg) are considered the most toxic HMs [47,48]. The following section will describe these four metals.

### 2.1. Cadmium

The main sources of this HM are mining activities and volcanic eruptions. The primary routes of cadmium (Cd) exposure are through the lungs and gastrointestinal tract, after which it is transported to the liver and kidneys—organs that are particularly susceptible to its toxicity. Prolonged exposure can lead to lung cancer [49]. Moreover, Cd causes itai-itai disease, which is associated with the consumption of Cd-contaminated water [50]. Furthermore, Cd also contributes to oxidative stress by negatively affecting antioxidant enzymes, leading to increased ROS levels. Inhibition of superoxide dismutase (SOD), catalase (CAT), and glutathione peroxidase (GPx) has been observed, correlating with elevated oxidative stress [51].

Available data indicate that cadmium may indirectly modulate the course of the Fenton reaction. Although this element does not directly catalyse the decomposition of hydrogen peroxide, it has the ability to displace physiologically relevant transition metals, such as iron or copper, from binding sites on proteins responsible for their transport or storage. This displacement increases the pool of free, redox-active forms of these metals, thereby enhancing their involvement in reactions that generate reactive oxygen species (ROS) and exacerbating oxidative stress.

In addition, cadmium disrupts cellular redox homeostasis through interactions with the antioxidant system, in particular with glutathione (GSH), one of the main non-enzymatic intracellular antioxidants [52]. Notably, mitochondria are also highly susceptible to the damaging effects of Cd. Increased expression of the pro-apoptotic protein Bax and decreased expression of the anti-apoptotic protein Bcl-2 has been reported. These changes in protein expression result in the activation of caspase-9 and caspase-3, which are key markers of cell apoptosis [53].

### 2.2. Arsenic

Arsenic (As) is known to be highly toxic to humans, especially in its inorganic form, which is classified as a carcinogen. The primary targets of As toxicity are the brain and liver [51]. As has been shown to generate ROS in various cell types, especially leukocytes, vascular smooth muscle cells, and vascular endothelial cells [54]. The production of superoxide radical anion (O2˙^−^), hydroxyl radical (˙OH), peroxyl radical (ROO˙), and singlet oxygen (^1^O_2_) has been reported. Moreover, the formation of nitrogen-derived free radical (RNS), such as nitric oxide (NO˙), has also been observed [55].

Exposure to As has been associated with decreased levels of SOD and CAT, as well as reduced mitochondrial membrane potential [56]. Oxidative stress induced by inorganic arsenic activate the Nrf2 (nuclear factor erythroid 2-related factor 2) transcription factor, which plays a key role in regulating the cell’s antioxidant response. Exposure to As leads to the induction of the Nrf2/HO-1 (heme oxygenase-1) pathway, a phenomenon which has been observed in various cell lines, including hepatocytes, keratinocytes and osteoblasts [57]. Activation of Nrf2 in these cells enhances HO-1 expression, resulting in increased production of biologically active metabolites.

The role of Nrf2 in the response to As toxicity remains ambiguous. On the one hand, Nrf2 exerts a protective effect through increased expression of antioxidant enzymes, thereby limiting oxidative damage. On the other hand, its prolonged activation may promote resistance to apoptosis, metabolic reprogramming and increased survival of cancer cells, potentially contributing to cancer development [58].

### 2.3. Lead

Lead (Pb) is present in significant quantities in the environment, mainly due to increasing human activities such as mining and fossil fuel combustion [59]. Pb is highly toxic, particularly to children and pregnant women. It accumulates mainly in the bones, kidneys, and liver [60]. Exposure to lead ions (Pb^2+^) results in dysregulation of the antioxidant system by disrupting the balance of enzymatic detoxification mechanisms. Pb^2+^ has a high affinity for thiol (-SH) groups in protein structures, leading to inhibition of key antioxidant enzymes such as glutathione peroxidase (GPx), superoxide dismutase (SOD), and catalase (CAT).

The ability of lead to form covalent bonds with sulphur atoms is due to the high polarity of both the sulphur and Pb^2+^ atoms, which favours the permanent binding of this metal to the functional groups of enzyme proteins. Inhibition of the aforementioned enzymes reduces the ability of cells to efficiently remove reactive oxygen species (ROS), leading to their accumulation and the development of oxidative stress. Studies in a mouse model have shown that inhibition of antioxidant enzyme activity in the liver strongly correlates with increased ROS production and elevated markers of oxidative stress, clearly indicating that lead contributes to the induction of redox imbalances of potentially cytotoxic significance [61].

In addition to interactions with enzymes, lead has been shown to bind to the thiol groups of cysteine in the reduced glutathione (GSH) molecule, which can potentially lead to the activation of toxic pathways and the disruption of the redox balance of the cell. Such interactions contribute to the weakening of antioxidant defence mechanisms and a marked reduction in the total antioxidant capacity of the liver—an organ that plays an essential role in the detoxification of xenobiotic compounds and the maintenance of the body’s metabolic homeostasis [47].

### 2.4. Mercury

Mercury (Hg) occurs in the environment in three forms: elemental, organic, and inorganic. The organic forms of Hg, especially methylmercury (MeHg) and ethylmercury (EtHg), are considered the most dangerous and are classified as non-essential metals for humans [59,62]. The primary target of MeHg is the central nervous system. MeHg has the ability to interact with several functional groups: -SH, -SeH (selenol), -COOH (carboxyl), -CONH_2_ (amide) and -NH_2_ (amino), which can lead to increased production of ROS and, consequently, enhanced oxidative stress.

Excessive generation of reactive oxygen species (ROS) can trigger the activation of multiple signalling pathways, with particular importance attributed to the pathway regulated by the Nrf2 (nuclear factor erythroid 2-related factor 2) transcription factor. Nfr2 plays a key role in the cellular response to oxidative stress through the induction of genes encoding antioxidant and detoxification enzymes [47].

MeHg has a high affinity for the thiol groups of GSH, with which it forms the GS-MeHg complex. The formation of this complex leads to a marked reduction in the concentration of free, biologically active GSH, resulting in increased oxidative stress and contributing to the neurotoxic effects of MeHg [63].

Findings from epidemiological studies and animal model experiments indicate that MeHg induces significant neuronal losses in the cerebellum—a structure responsible for the control of movement and balance—and in the visual cortex, which is involved in the processing of visual information. Neuronal degeneration in these areas is closely linked to decreased GSH levels. The high millimolar concentration of glutathione in the mammalian cerebellum suggests that MeHg-induced oxidative stress results from both the direct generation of ROS and the direct interaction of MeHg with the cell’s antioxidant system [64].

In addition, MeHg adversely affects the activity of selenoenzymes such as glutathione peroxidase (GPx) and thioredoxin reductase, which contain selenium atoms at their active centres. The GPx/GSH system is one of the key defence barriers against oxidative stress. Inhibition of this system by MeHg impairs the detoxification of hydrogen peroxide (H_2_O_2_) and lipid peroxides. The accumulation of H_2_O_2_ promotes secondary generation of ROS, including hydroxyl radicals (-OH) via Fenton reactions involving Fe^2+^ or Cu^+^ ions, as well as peroxyl radicals. The reactive oxygen species formed show high reactivity towards biological structures, resulting in damage to membrane lipids, proteins and genetic material [65].

## 3. Effects of Black Tea Components on Heavy Metal Toxicity

In this review we focused on the experimental studies that have investigated the effects of TFs and TRs in cell culture models as protective compounds against HM toxicities. Potentially eligible studies were identified by searching Pubmed and Scopus.

The following search terms were used: [(heavy metals) OR (cadmium) OR (arsenic) OR (lead) OR (mercury)] AND [(in vitro) OR (cell culture)] AND [(theaflavins) OR (TF) OR (thearubigins) OR (TR) OR (black tea)]. An additional manual search was also conducted by reviewing the reference lists of relevant papers.

In the available literature, it is difficult to find reports on the effects of TFs or TRs on HM toxicity in in vitro studies. Although both compounds have been reported to show strong antioxidant properties, this area remains under-researched and requires in-depth analysis. However, a study by Shon and colleagues [66] describes the protective effects of TFs and TRs on COS-7 cells (monkey kidney fibroblasts) exposed to copper (500 µM) and cadmium (5 µM) for 24 h. Pre-treatment with 10, 20, 40 and 80 µg ml^−1^ of TFs and TRs resulted in increased cell viability, together with an increase in concentration of TFs and TRs. Additionally, hydroxyl radical scavenging activity and protection from apoptosis and damage caused by oxidative stress were also observed.

Alternatively, several studies have investigated the interaction of heavy metals with black tea extract. Mukherjee et al. [67] observed a reduction in the cytotoxic effects of chromium and arsenic in bone marrow cells in mice following black tea administration. A 6-day treatment with black tea infusion, simulating human consumption and administered by gavage twice daily, resulted in a significant reduction in chromosomal damage caused by a single dose of these heavy metal salts.

Furthermore, Areba and colleagues reported that black tea extract exhibits protective properties against cadmium toxicity in brain tissue. Their study was conducted on brain tissue from healthy Wistar rats that were co-administrated with Cd (2 mgkg^−1^ bw/day) and black tea extract (400 mgkg^−1^ bw/day). The analyses revealed decreased levels of GSH and the lipid peroxidation marker TBARS, indicating improved antioxidant defence. The authors concluded that tea extracts significantly reduced the toxicity of cadmium salt and had a positive impact on the antioxidant defense system [68].

## 4. Conclusions

Human exposure to the negative effects of heavy metals is increasing, primarily due to the progressive development of industry—one of the main sources of heavy metal contamination. As a result, effective strategies to protect the body against heavy metal toxicity and its associated negative effects are constantly being sought. A great deal of attention has already been devoted to research on the protective effects of EGCG from green tea against the oxidative stress induced by heavy metals. However, black tea is significantly more popular than green tea worldwide.

It is hypothesised that the polyphenols theaflavins (TFs) and thearubigins (TRs) present in black tea may have stronger antioxidant properties than EGCG due to structural differences. Although research is limited, black tea polyphenols have shown protective effects against heavy metal toxicity.

This literature review highlights that research on the antioxidant potential of black tea polyphenols in the context of heavy metals toxicity is still underdeveloped. It is clear that this is a topic requiring further extensive analysis, particularly in the case of TRs, for which there is a notable gap in research and literature concerning the protective potential of this compound on cells exposed to heavy metals.

## Figures and Tables

**Table 1:** Differences in black and green tea composition [1,7]. Compounds in bolds shows the main differences between black and green tea.

	Concentration [%]	
Compounds	Green Tea	Black Tea
**Catechins**	**30–35**	**3–10**
Simple polyphenols	2	3
**Oxidised polyphenols**	**6**	**23–25**
Flavonols	2	1
Theanine	3	
Aminoacids	3	
Peptides	6–16	
Lipids	2–8	
Carbohydrates	10–15	
Caffeine	3–6	
Minerals	4–10	
Pectins	3–4	
Chlorophyll/other pigments	0.5	
Volatile compounds	0.01	

## Data Availability

Not applicable.
